# Practical Tools and Guidelines for Young Oncologists From Resource-Limited Settings to Publish Excellence and Advance Their Career

**DOI:** 10.1200/GO.21.00310

**Published:** 2021-12-15

**Authors:** Khalid El Bairi, Ouissam Al Jarroudi, Said Afqir

**Affiliations:** ^1^ Department of Medical Oncology, Mohammed VI University Hospital, Oujda, Morocco; ^2^ Faculty of Medicine and Pharmacy, Mohammed Ist University, Oujda, Morocco

## Abstract

Cancer research is evolving worldwide. However, publishing high-quality academic literature in oncology remains challenging for authors in the developing world. Young oncologists in low- and middle-income countries experience several barriers including lack of funding and research facilities, as well as inadequate training. Publication best practices, science integrity, and ethics are required to improve oncology research quality and therefore, improve patients' care in these countries. To achieve this goal, we propose some basic principles and tools that may help young oncologists especially in developing countries overcome these issues and boost their academic careers.

## INTRODUCTION

Research in oncology is a highly active field with more than 1 million papers published in the past 5 years alone, as clearly shown by the US National Library of Medicine Pubmed/Medline database.^1^ Notably, the largest part of this progress has been achieved in high-income countries. By 2030, it is expected that cancer will be the primary cause of death in low- and middle-income countries (LMICs).^2^ Promisingly, there is an enthusiastic movement among these developing countries to enhance scientific production in this field, but it is still facing big challenges, particularly regarding the shortage of the oncology workforce. Moreover, health researchers in these settings are markedly under-represented and there is a significant inequity regarding publications in leading medical journals compared with authors from high-income countries.^3-5^ Several well-documented factors have an influential impact on the poor production of oncology research papers in developing countries, including barriers to accessing oncology literature, inadequate training in research, lack of or poor mentorship, and rare international partnerships.^5,6^ Moreover, these authors are under-represented because of the competition of well-trained researchers from high-income countries and low financial support allowed for research in their affiliations. There is also a growing attitude among these authors to publish their cancer research with predatory publishers instead of reputed journals.^7,8^ Therefore, young oncologists in these countries should have access to optimal guidance as they might have a significant contribution to the global knowledge pool in oncology. Improving publication quality requires ethical standards and basic principles, as well as research integrity good practices. In this perspective, we discuss these critical emerging issues together and provide some practical principles that early-career cancer researchers from developing countries have to follow to improve the quality of their research. This is anticipated to potentially advance their academic expertise. We believe that this paper will provide useful guidance for curricular development in these settings with limited resources, and therefore, improve cancer control.

**CONTEXT**

**Key Objective**
To provide a useful research toolkit to young oncologists in under-resourced settings.
**Knowledge Generated**
Curricular development in countries with limited resources needs guidance for a better cancer research that affects patients' outcomes. A number of rules and principles can be followed to improve research reporting in low-income settings.
**Relevance**
The tools and guidelines discussed in this paper will certainly help young cancer researchers developing skills for a better career in oncology.

## PRACTICAL TOOLS AND GUIDELINES

### Always Get Approval From an Ethics Committee Before Conducting a Study Involving Human Participants

Lack of ethics committee approval is one of the main limitations for cancer research of authors in LMICs. Researchers should get prior approval for their studies to get more chances of acceptance in reputed oncology journals. Clinical and biomedical researches that include animal models and human participants require an earlier ethics committee approval before starting the study enrollment. According to the Declaration of Helsinki of the World Medical Association, research on human subjects should be clearly formulated in experimental protocols and these should be submitted to independent ethical review boards (ethics committees and institutional review boards) for approval before the study starts. Notably, every participant has to be clearly informed about the different aims, funding sources, and potential conflicts of interest, and also the significant anticipated benefits for advancing the related scientific field before giving their written consents to participate.^9^ Moreover, a detailed description of the potential risks related to interventions should be explained to participants to limit the possibility of causing any harm and to avoid any future legal actions. In countries with unavailable ethics committees, participant's written consents should be provided and the manuscript cover letter to handling editors should mention the difficulty of achieving a prior ethics committee approval. Importantly, cancer researchers should store these signed consents in print and electronically for future requests from journal editors. In exceptional situations where consents would be difficult to obtain, data collection from participating subjects may be conducted only after approval of a research ethics committee. If an author had no ethical committee available for consultation in their setting, they can request other country-based ethical review boards to review their protocol. Moreover, if the authors have no formal approval, the reason why should be explained in the methods section and also in the cover letter for the journal editor. For transparency, during manuscript writing, the name and the location of the ethical committee that approved the research protocol and the related approval number should be provided in the methods section. Infringement of these rules may cause serious damages to researchers, including retraction of publications after peer review. A useful nonexhaustive list of ethical committees can be found in the WHO portal.^10^ For more reading, see Refs. 11,^12^.

### Register Your Study Protocol

Study protocols have a cornerstone place in published reports and are decisive components of research. Their design occupies a central role in providing accurate data when testing hypotheses. Prospective registration of study protocols is becoming essential to enhance credibility, reproducibility, and transparency for both observational and interventional studies.^13,14^ In addition, meta-research studies, including systematic reviews with or without meta-analyses, are potential article types that require prior registration on online databases such as International Prospective Register of Systematic Reviews, which have a significant positive impact on research results by increasing review quality.^15^ Several initiatives that support this transparency movement have been developed and have created various databases for this purpose, encompassing free and paid services (Table 1). Web-based sources such as the American ClinicalTrials.gov database, which is maintained by the National Library of Medicine at the National Institutes of Health, are a good example of a successful free service that provides updated data on human studies for researchers, clinicians, and also for patients and their family. Information related to clinical trial designs and result dissemination are updated during the study progress. The Pan African Clinical Trials Registry,^16^ which is the first WHO-recognized registry of clinical trials in Africa, is a comprehensive database to freely register and search clinical trials for all researchers of the African continent as recommended by the requirements of the International Committee of Medical Journal Editors. This initiative assists researchers understanding African research features and allows researchers to identify research gaps for future clinical investigations.^17^ These databases adhere to local policies, legal obligations, and ethical obligations of findings disclosure globally. Therefore, their use is recommended for all cancer researchers before the beginning of enrollment and data collection.

**TABLE 1:**
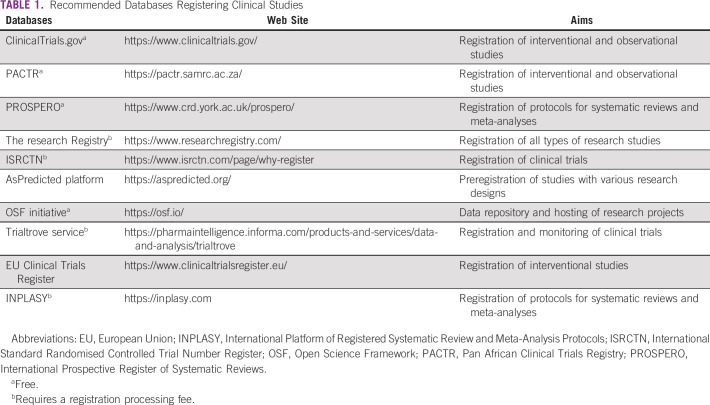
Recommended Databases Registering Clinical Studies

### Follow Consensus Reporting Guidelines When Conducting and Publishing Research

Cancer research (particularly systematic reviews and observational and interventional clinical trials) has a remarkable influence on clinical practice guidelines. The relevance and quality of cancer research is widely judged by its final published reports. In the era of publish or perish, the usability and actionability of oncology research findings can be damaged by their poor reporting. It is commonly noticed that reports of health research may omit crucial information that should be provided to support the authors' hypotheses and limit reporting biases.^18,19^ This may dramatically mislead clinicians and researchers as well as decision makers when managing patients with cancer. These misleading research results are avoidable by a proper use of reporting guidelines. In this perspective, authors should provide accurate and complete reporting of their rationale, methodology, results, their significance for practice, and limitations to maximize objectivity and extrapolation for daily management of patients with cancer. Several reporting guidelines have been developed according to the design of the studies to enhance the completeness and transparency as well as clarity of health research. Enhancing the QUAlity and Transparency Of health Research (EQUATOR) network^20^ is a valuable resource for researchers that was developed by several editorial working groups and provides a large and robust database of more than 400 reporting guidelines.^21^ This freely available and comprehensive searchable library provides explanations, online training, and guidance for use in several languages to help authors report their findings responsibly. Moreover, the database site contains toolkits for writing and peer-reviewing research for anyone involved in scholarly publishing. Recently, the EQUATOR has developed EQUATOR Oncology that aims to support cancer researchers for high-quality reporting of their research and is recommended for all young oncologists working in clinical investigation.^22^ An illustrative example of these guidelines is the Reporting Recommendations for Tumor Marker Prognostic Studies (REMARK) that was developed to address the common issues in studies reporting findings on cancer biomarkers for predicting prognosis.^23^ The REMARK checklist consists of 20 items that should be considered when designing, conducting, and writing manuscripts that describe tumor markers. However, despite the implementation of EQUATOR guidelines widely by cancer researchers and journals, they are frequently used inappropriately by authors.^24-26^ Therefore, young oncologists are invited to properly accomplish training on how to use them in their research on the basis of EQUATOR tools before writing their manuscripts for publication. Table 2 provides a nonexhaustive list of reporting guidelines to be used when writing research.

**TABLE 2:**
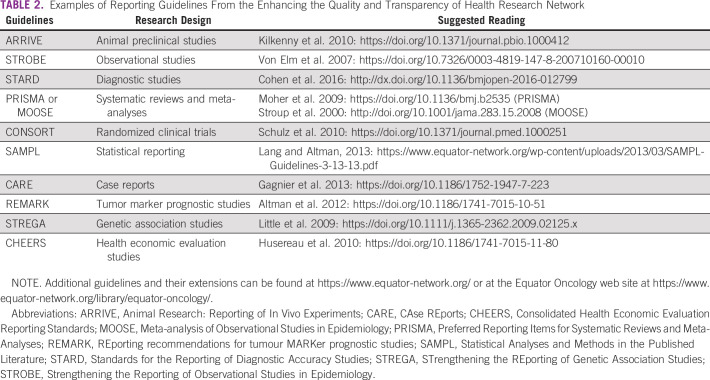
Examples of Reporting Guidelines From the Enhancing the Quality and Transparency of Health Research Network

### Learn the Basics of Evidence-Based Medicine and Biostatistics

The recent development seen in all areas of health care is associated with the rapidly growing field of medical publishing. Clinicians are therefore faced with a huge number of published articles in their field, which makes their use in health care decisions difficult. The so-called evidence-based medicine (EBM) was developed to systematically appraise and apply the current evidence in the context of patients' condition. EBM is a cornerstone of our daily practice, and this area of clinical research methodology is highly active in oncology. The use of EBM principles in daily oncology care is expected to improve outcomes of patients with cancer. EBM is founded on the critical evaluation of the findings of randomized and controlled clinical trials, their meta-analyses, as well as other study types. Translating the findings of EBM in oncology into our real life is challenging. During residency in medical oncology and related specialties, courses on developing skills in EBM are rarely included in the regular training, particularly in the context of countries with limited resources. Thus, enhancing EBM skills in the training of young oncologists is highly recommended. Several strategies can be adopted, particularly, online-based courses and support. The use of the online COCHRANE training resources is highly recommended to master this area by young oncologists. COCHRANE offers various online courses, a rich documentation on EBM, as well as other tools that can be used for this purpose (see Appendix Table A1 and Table 3). Other essential guides such as the Journal Club of the European Society for Medical Oncology (ESMO) young oncologists' corner^27^ and the ESMO Handbook of Interpreting Oncological Study Publications^28^ are good examples of successful tools for young oncologists that desire to be involved in evaluating evidence and learning the basics of evidence-based oncology. The ASCO training resources and its e-learning platform^29^ are also beneficial for developing capacities when doing EBM. In addition, the BMJ Best Practice tools and checklists^30,31^ can also be useful when reviewing the evidence on particular topics.

**TABLE 3:**
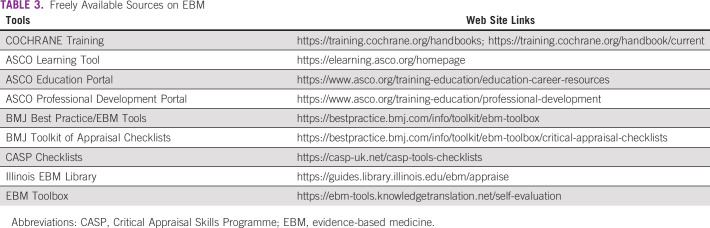
Freely Available Sources on EBM

In several oncology publications, statistical reporting is incomplete and important interpretable and actionable data are not provided. Therefore, young researchers should support their manuscripts by a detailed description of statistical approaches used to test their hypotheses as recommended by international EQUATOR-related guidelines. The Statistical Analyses and Methods in the Published Literature guidelines were developed to help scientists report all the required statistical methods for publication in academic journals.^32^

Young oncologists should also learn how to perform basic statistical testing such as associations, correlations, logistic regression, Kaplan-Meier estimation, and especially the Cox hazard proportional model, as this statistic is widely used in cancer research to study time to event data. This will allow them to perform good oncologic studies in their setting with a focus on survival analysis and its associated predictors. They are also encouraged to consult statisticians when writing the design of their studies and also before data collection. This is crucial for testing hypotheses as the expected findings are associated with the initial sample size considerations and the design used. Useful publications and guides on how to understand clinical biostatistics with a particular focus on oncology can be found in Table 4.

**TABLE 4:**
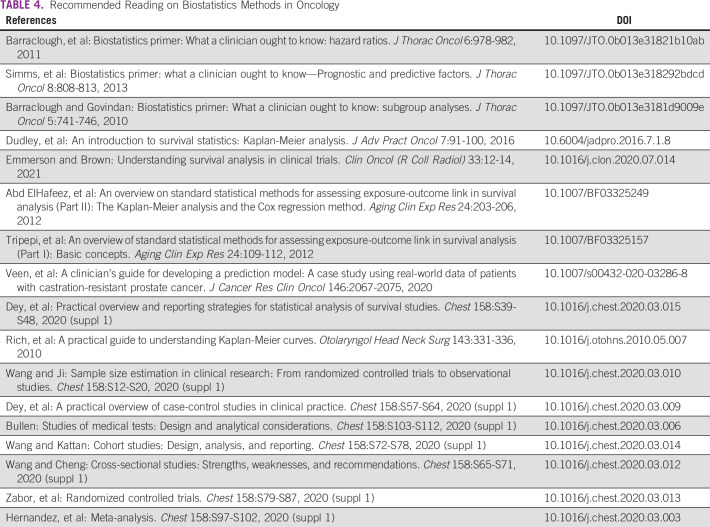
Recommended Reading on Biostatistics Methods in Oncology

### Do Not Publish in Predatory Journals

An impressive number of open-access predatory journals and publishers are launched every year as money-collecting machines. They are now well known by their features such as the absence of peer-review, plagiarism tolerance, misleading US-based addresses, confusing indexing and fake high-impact factors, spam invitations, the nearly 100% acceptability rates, fake and unqualified editors, and so on.^33,34^ They basically accept everything without any verification of the contents of the submissions. With their typical e-mails, predatory journals and fake conferences will invite authors to submit their research. In addition, they use attractive names for their fake journals including the terms Canadian, British, American, European, International, etc, to attract the attention of potential authors. These authors, particularly from developing countries with limited training in publishing ethics and under pressure to publish faster, submit their findings intentionally or unintentionally to predatory journals to boost academic promotion, grant applications, and jobs. This issue has become a bullying crisis that threatens the scientific integrity of research findings. This causes severe damages for young authors in these settings as these for-profit journals publish without proper peer review and therefore their articles are questionable, not credible, and have little scientific impact.^35^ It is mandatory that oncologists avoid these predatory journals and not accept to be part of their editorial boards. Unfortunately, it has been recently noticed that predatory journals can infiltrate respectable indexing/abstracting databases.^8^ Therefore, authors should be vigilant when selecting journals for publication. They must always take the needed time to verify the quality of academic journals before submitting their research. Several criteria can be used to find the most suitable journal for a manuscript. This includes multiple indexing on the three recognized databases—Medline, Scopus, and Web of Science—as this may limit the presence of infiltrating predatory journals. The use of the Beall's list can also be useful, but it is not regularly updated as dozens of new predatory journals are launched every month.^36^ The prestige and the experience of trusted publishers should also be considered as it is rare to have a predatory journal published by a standard academic publisher. Appendix Table A2 shows trusted publishers that place nonfraudulent information regarding indexing/abstracting, impact factor and other metrics, and data on their official web site.

Some measures can be considered by authors to better repair this emerging issue. When their research findings are published accidentally in a predatory journal, they should deposit their article for evaluation by PubMed Central. This database has an initiative to index articles after an internal peer review and therefore, this is highly recommended.^37^ For more transparency and credibility, articles published in predatory journals can be deposited into social networks such as ResearchGate to enhance postpublication open peer review with the scientific community. At an individual level, authors are compassionately invited to explain why they published their research in predatory journals in their curriculum vitae to enhance the reliability of their academic achievements.^38^ Their research institutions should also publicly discuss this matter to prevent any other predatory publications in the future (for recommended reading on this topic, refer Appendix Table A3).

### Participate in the Open Science Movement

Open access refers to making publication contents freely and openly accessible for readers and authors for reuse under the CC BY 4.0 license.^39^ The broader term open science is used for a more general movement to disseminate knowledge and improve collaboration between researchers and the public. This framework has been developed worldwide by funders, policymakers, and research institutions to enhance access to research findings. From this perspective, cancer researchers with limited resources may benefit from the *Research4Life* partnership initiative that enables access to peer-reviewed content via a unique public-private partnership between United Nations Agencies, Yale and Cornell Universities, and academic publishers. Participating journals may provide partial or full waivers of article-processing charges according to the World Bank classification. Thus, authors should check the web sites of open-access journals when desiring to publish this way. However, the classification criteria to benefit from this advantage may be deceptive in some cases. As Moroccan young cancer scientists, we can benefit from a 50% waiver but we cannot afford it. Open science has a financial toxicity for oncologists from developing countries as their publications are rarely funded. Therefore, green open access^40^ should be supported and offered to these authors. Some other national projects such as the publication subsidy (*Programme de Subvention des Publications*) by the *Institut de Recherche sur le Cancer* in Morocco that supports its research associates to pay the charges of open access is a good program that should be developed to progress open science in similar settings. When choosing a subscription-based journal, submitting the draft to preprint servers such as Research Square and medRxiv is recommended for data sharing widely to enhance prepublication and postpublication peer review as well as the open science initiative. However, authors must check the policy of target journals for submission as some of them do not accept such deposition in preprint servers. Recommended preprint servers to be used to enhance transparency and publishing ethics can be found in Table 5. Importantly, there is a disparity related to the access to medical literature between LMICs and developed countries.^41-44^ In keeping with this, several initiatives were developed to provide access to hidden content behind paywalls, including the questionable Sci-Hub project. This service offers free access to a great pirated proportion of articles.^45-47^ At the same time, licensed and authorized services also exist and can be used by authors from LMICs and those with limited resources to sustainably access research publications. For instance, EndNote Click (formerly Kopernio) is an online tool that can be used by researchers worldwide to search for free articles–related portable document format.^48^ This full-text finder service can be integrated into web browsers to identify freely available article text.^49,50^ Other new tools such as Open Access Button^51^ and Unpaywall^52^ with their advantages and disadvantages were nicely discussed elsewhere.^50^

**TABLE 5:**
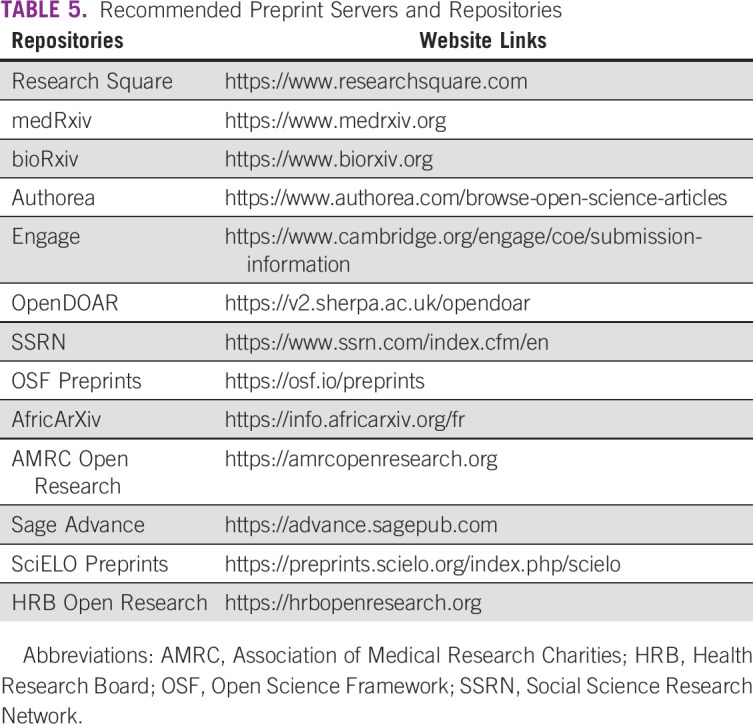
Recommended Preprint Servers and Repositories

### Get Involved in Working Groups and Scientific Social Networking

The use of scientific social networking has recently emerged as a potential approach for professional development and collaboration.^53^ Integrating social media in modern oncology practice and research is a recent trend that enabled the expansion of working groups to improve patients' care and foster research between oncologists from low-income countries and others from prestigious institutions from the developed world.^54^ This opportunity provides an extremely promising frontier for cancer research without physical barriers. Moreover, this allows easy interaction and communication between oncologists worldwide to enhance second opinions on difficult patients' cases. In this perspective, *The ONCOLLEGE* working group^55^ is a good example of ideas sharing and collective contribution of young oncologists to improve cancer patients' care via the use of social media to discuss cases that need second opinions. This initiative developed by youth working in different fields of oncology has also facilitated the development of several research projects that resulted in publications gathering international authors together.^56-60^ Notably, The International Immuno-Oncology Biomarker Working Group on Breast Cancer played a pivotal role in bringing clinicians and scientists to work together and improve cancer care through cooperation.^61^ This working group has published several impactful papers on the implementation of tumor-infiltrating lymphocytes in settings with limited resources as a surrogate for programmed death-ligand 1 testing in breast cancer.^62-66^ Other similar works and initiatives were also successful in providing practice changes through networking.^67^ These conceptual models are valuable for integrated care delivery and interorganizational collaboration.^68^ Despite these ideas being in early proof-of-concept stages, young oncologists are encouraged to enthusiastically participate in this movement to boost their career. Oncology is an active and a rapidly evolving field that requires collaboration and taking an active role in shaping its future. Moreover, peer networking and regional collaboration between oncologists in LMICs should also be considered to develop research affecting outcomes as they share similar issues related to cancer control. This will certainly help establishing durable long-term research initiatives.

Memberships in international oncologic societies such as ESMO and ASCO is highly recommended as they offer various travel grants, training programs, and support for oncologists to better manage patients with cancer and advance research on cancer. Interested oncologists can benefit from free or low fees of membership and enjoy various benefits to make their career in oncology better. A recommended list of working groups, international and recognized cancer organizations, and societies can be found in Appendix Table A4. They can be consulted regularly to explore education and training opportunities for young oncologists.

### Apply for Funding Opportunities, Fellowships, and Grants

Cancer research in countries with a high income is predominantly sponsored by the pharmaceutical industry that addresses financial interests. Moreover, funding agencies play an important role in the performance of research institutes in these settings. The development of these human and funding capacities in high-income countries has generated outstanding improved outcomes that we have seen in the past few years. In LMICs, as mentioned above, lack of skilled researchers and motivation to conduct research as well awareness of the impact of the real-world studies are the significant factors affecting cancer research.^5,6^ Financial barriers for conducting clinical trials and other types of oncology research are additional factors that are negatively associated with the poor productivity of scientific publications that affect patients' outcomes in under-resourced settings.^5,6^ Promisingly, various initiatives are available to authors in LMICs to fund their research and boost their career. This encompasses fellowships, grants, and other funding opportunities from international organizations and scientific oncology societies. A good and illustrative example is the Conquer Cancer, an ASCO foundation, which offers several funding opportunities and awards to cancer researchers that cover various cancer settings for oncologists from all over the world. To benefit from these funding projects, oncologists from LMICs should apply for memberships as this is a requirement for submitting their proposals. A useful list of web site links to these opportunities can be found in Table 6. Importantly, most of these funding agencies require a prior writing of grant research proposals. Therefore, young oncologists should have the skills and guidance for successful research grant and fellowship applications. In this regard, various papers addressing recommendations for grant and research proposal writing were published to guide these scientists.^69-77^ Notably, this also usually needs English language skills when writing proposals. English has long been the dominant language in scientific writing and publishing. Nearly all oncology journals publish in English, and those with other languages such as French are currently switching to it. Learning English and scientific writing earlier in the career of oncologists is anticipated to create the environment for fostering international collaboration and publishing excellence. Native languages should be reserved for the popularization of science and are needed for communication with the public as well as patients with cancer. Useful links for developing scientific writing skills can be found in the Equator Network initiative.^78^

**TABLE 6:**
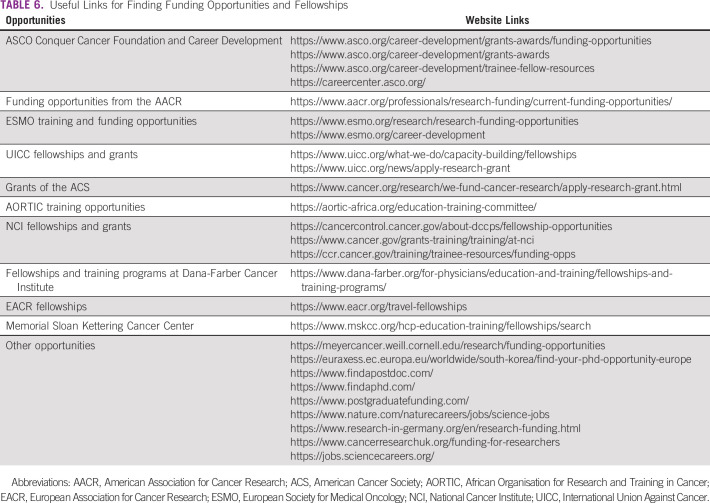
Useful Links for Finding Funding Opportunities and Fellowships

### Learn Peer Review

The peer-review system plays a central role in maintaining the high standards of scientific research and academic publishing. This process evaluates research and literature-based findings for originality, significance, quality, and impact on clinical practice.^79,80^ The duration of peer review varies significantly between publishers and academic journals and may be lengthy. This very long process is because of the voluntary aspect of peer reviewers and associate editors. Access of early-career oncologists to peer review is challenging as training to develop this expertise is not included in their residency and research programs. Moreover, reviewing requires mentorship, specific instructions, experience in publishing, biostatistics, and clinical research methods. Currently, several web-based initiatives and resources were developed to help young investigators and are accessible worldwide for free (Appendix Table A5). A good example of these opportunities is the Publons Academy certified training program.^81^ This platform aimed to train young scientists the essential skills required for peer review by 10 modules and active mentorship by a qualified reviewer and scientific experts in the field of the participants. This is expected to foster the career of young oncologists to build proficient talents in their research field. Skilled peer reviewers may be invited by journals to be associate editors. This is a required competency for those desiring to develop academic journals for their local settings.

### Do Not Be the Last to Know About the Latest News in Cancer Research

Oncology research is evidently a greatly active field with remarkable interventional and observational clinical trials that release on-fire findings every week. This outstanding cancer research has a powerful and direct impact on patients' survival and outcomes. Summary and expert discussion on practice-changing clinical research is provided by various trusted educational platforms and companion web sites of international societies and organizations that were created to deliver the latest news in oncology. A number of these services can be found in Appendix Table A6. Educational portals for oncologists, such as The ASCO Post, the ESMO News/OncologyPRO, the ecancer, and also the ASCO Communications, which provide oncology news allow readers to be up to date with the latest developments that influence daily practice by newsletters and interacts with their authors. An illustrative example of these good free online initiatives is ecancer.^82^ This resource presents updated and free-of-charge knowledge on all areas of cancer research to meet global oncology needs. Ecancer publishes open research and research news, shares video resources and insights from covered conferences, provides continuing medical education–accredited e-learning in multiple languages, and provides free or low-cost in-person educational training across the globe. This initiative also offers education for patients with cancer through its program *ecancerpatient.org* intended to simplify oncology news and information in a friendly format.

### Be Involved in Blogging, Volunteering, and Simplifying the Oncologic Sciences for the Public and Undergraduate Students

Blogging in oncology is a relatively recent extracurricular activity. It permitted to several oncologists and patients' advocates individual sharing of authors' perspectives and opinions on emerging concerns in oncology such as access to treatments, financial toxicities of novel drugs, governmental policies, supportive care, and issues in clinical trials that are not covered by traditional peer-reviewed academic journals. Blogging has a potent impact on improving patients' care by attracting a general audience that may increase awareness of current issues in oncology globally. Various platforms for blogging are available for all oncologists worldwide to share their ideas and interact with the oncology community. The ASCO Connection is a good example of a free successful blogging platform that can be used by early-career oncologists to issue their thoughts.^83^

Volunteering in offering care, patients' education, preventive consultations, and popularization of cancer sciences provide an opportunity for young oncologists to build leadership and soft skills. Therefore, these future leaders will expectedly be competent at accurately speaking to the public and attracting decision makers and policymakers to respond to the needs of their oncology settings. Importantly, a shortage of cancer specialists is an emerging issue that needs to be addressed in developing countries. Involving undergraduate medical students in cancer research projects earlier during their career is a good initiative to attract them to this stunning specialty. Furthermore, attracting more students to oncologic sciences to prepare the future workforce can be achieved by early exposure to these specialties during their graduate training.^84^ Notably, organizing courses on the basics of oncology for medical students to enhance the scientific interaction with senior oncologists seems to be promising.^85^ Oncology is a rapidly evolving field in which they can practice EBM and research compared with other medical specialties. Also, national societies should engage these students in internships and courses to make a career in oncology to boost the number of oncologists and respond to the increasing need for skilled care physicians.

## CONCLUSION

Authors in countries with limited resources doing research on this pivotal medical field still need training and support to advance cancer research in their settings. To date, little is known about the capacity of cancer researchers, including oncologists in conducting accurate research to meet the needs for appropriate studies and actionable data for cancer burden control. Further bibliometric investigations on research outputs of oncologists from LMICs are required and awaited. This will hopefully bridge the gap of clinical research that affects patients' outcomes in these settings. Moreover, this will address the current challenges that health care professionals face in these countries by implementing better evidence-based programs and recommendations for best practices. We hope that this paper will provide useful information and guidance for cancer researchers from developing countries to boost their career.

## Figures and Tables

**TABLE A1:**
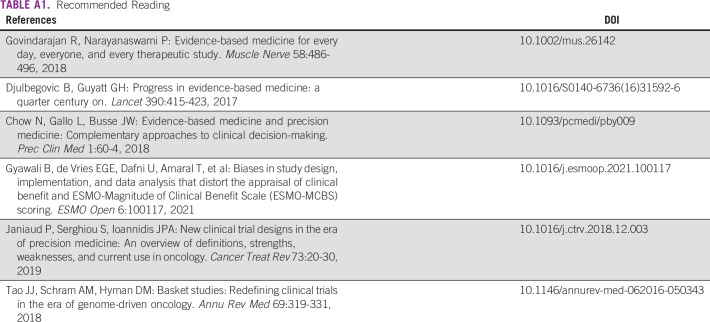
Recommended Reading

**TABLE A2:**
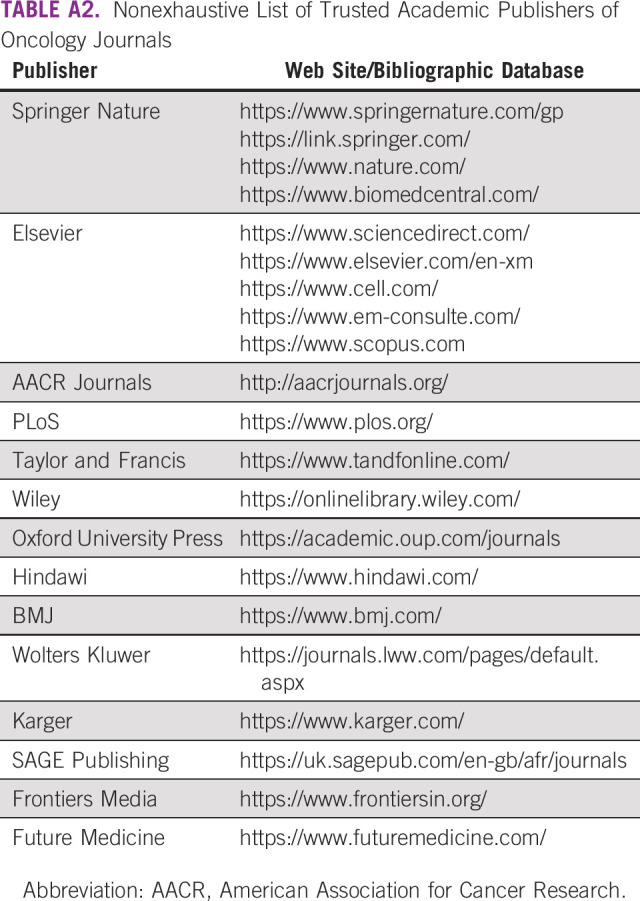
Nonexhaustive List of Trusted Academic Publishers of Oncology Journals

**TABLE A3:**
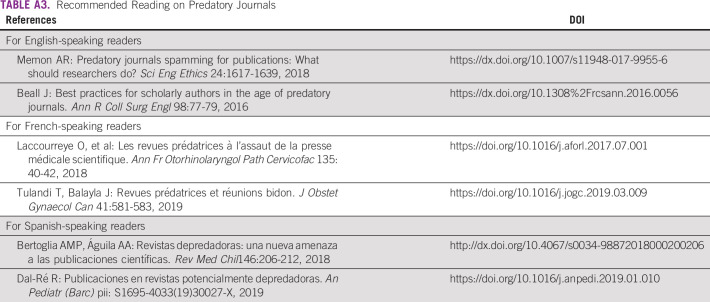
Recommended Reading on Predatory Journals

**TABLE A4:**
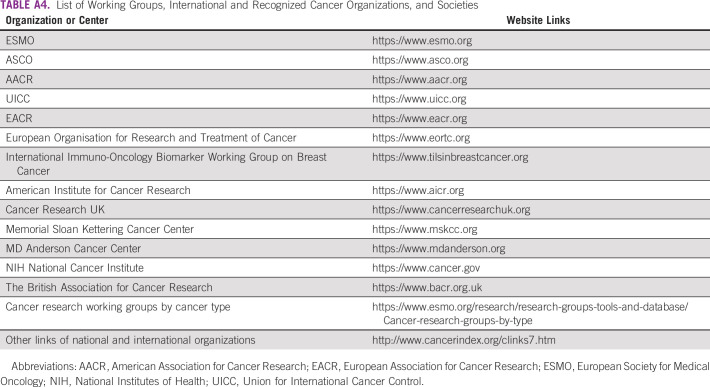
List of Working Groups, International and Recognized Cancer Organizations, and Societies

**TABLE A5:**
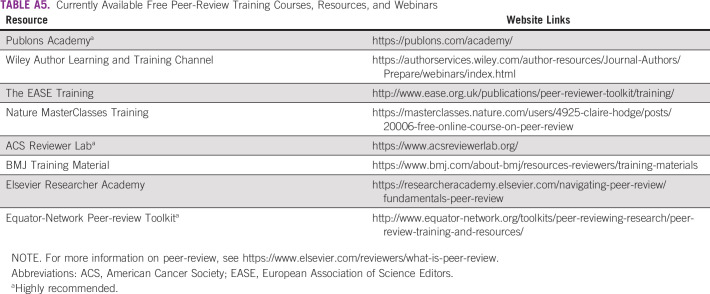
Currently Available Free Peer-Review Training Courses, Resources, and Webinars

**TABLE A6:**
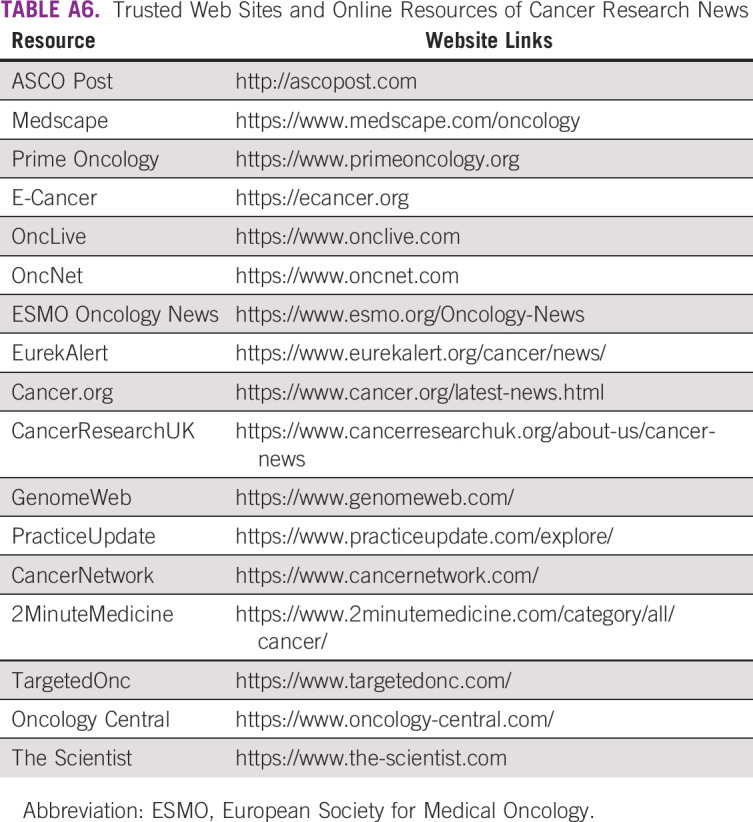
Trusted Web Sites and Online Resources of Cancer Research News

## References

[1] National Library of Medicine: https://pubmed.ncbi.nlm.nih.gov/10.1080/15360280801989377 28792816

[2] Bray F Jemal A Grey N et al: Global cancer transitions according to the Human Development Index (2008-2030): A population-based study. Lancet Oncol 13:790–801 2012 22658655 10.1016/S1470-2045(12)70211-5

[3] Sumathipala A Siribaddana S Patel V: Under-representation of developing countries in the research literature: Ethical issues arising from a survey of five leading medical journals. BMC Med Ethics 5:E5 2004 15461820 10.1186/1472-6939-5-5 PMC524359

[4] Merriman R Galizia I Tanaka S et al: The gender and geography of publishing: A review of sex/gender reporting and author representation in leading general medical and global health journals. BMJ Glob Health 6:e005672 2021 10.1136/bmjgh-2021-005672 PMC8118011 33986001

[5] Alemayehu C Mitchell G Nikles J: Barriers for conducting clinical trials in developing countries—A systematic review. Int J Equity Health 17:37 2018 29566721 10.1186/s12939-018-0748-6 PMC5863824

[6] Barrios CH Mano MS: Is independent clinical research possible in low- and middle-income countries? A roadmap to address persistent and new barriers and challenges. Am Soc Clin Oncol Ed Book 41:1–10 2021 10.1200/EDBK_321335 33830826

[7] Forero DA Oermann MH Manca A et al: Negative effects of “predatory” journals on global health research. Ann Glob Health 84:584–589 2018 30779504 10.29024/aogh.2389 PMC6748305

[8] Severin A Low N: Readers beware! Predatory journals are infiltrating citation databases. Int J Public Health 64:1123–1124 2019 31342093 10.1007/s00038-019-01284-3

[9] Schroter S Plowman R Hutchings A et al: Reporting ethics committee approval and patient consent by study design in five general medical journals. J Med Ethics 32:718–723 2006 17145913 10.1136/jme.2005.015115 PMC2563342

[10] World Health Organization: National ethics committees database. https://apps.who.int/ethics/nationalcommittees/nec.aspx

[11] Mpolesha JMK Mapatano MA Elzawawy A et al: Ethics of conducting cancer research in developing countries Stefan D Cancer Research and Clinical Trials in Developing Countries. Cham, Switzerland Springer 2016

[12] World Medical Association: WMA Declaration of Helsinki – ethical principles for medical research involving human subjects. https://www.wma.net/policies-post/wma-declaration-of-helsinki-ethical-principles-for-medical-research-involving-human-subjects/19886379

[13] Dal-Ré R Ioannidis JP Bracken MB et al: Making prospective registration of observational research a reality. Sci Transl Med 6:224cm1 2014 10.1126/scitranslmed.3007513 24553383

[14] Zwierzyna M Davies M Hingorani AD et al: Clinical trial design and dissemination: Comprehensive analysis of clinicaltrials.gov and PubMed data since 2005. BMJ 361:k2130 2018 29875212 10.1136/bmj.k2130 PMC5989153

[15] Sideri S Papageorgiou SN Eliades T: Registration in the international prospective register of systematic reviews (PROSPERO) of systematic review protocols was associated with increased review quality. J Clin Epidemiol 100:103–110 2018 29339215 10.1016/j.jclinepi.2018.01.003

[16] Pan African Clinical Trials Registry: https://pactr.samrc.ac.za/

[17] Abrams A Siegfried N: The Pan African Clinical Trials Registry: Year one data analysis of the only African member of the World Health Organization Network of Primary Registries. J Evid Based Med 3:195–200 2010 21349070 10.1111/j.1756-5391.2010.01099.x

[18] Glasziou P Altman DG Bossuyt P et al: Reducing waste from incomplete or unusable reports of biomedical research. Lancet 383:267–276 2014 24411647 10.1016/S0140-6736(13)62228-X

[19] Glasziou P Meats E Heneghan C et al: What is missing from descriptions of treatment in trials and reviews? BMJ 336:1472–1474 2008 18583680 10.1136/bmj.39590.732037.47 PMC2440840

[20] Equator Network: https://www.equator-network.org/

[21] Gould KA: The EQUATOR network: A resource for authors. Dimens Crit Care Nurs 35:350 2016 27749439 10.1097/DCC.0000000000000213

[22] MacCarthy A Kirtley S de Beyer JA et al: Reporting guidelines for oncology research: Helping to maximise the impact of your research. Br J Cancer 118:619–628 2018 29471308 10.1038/bjc.2017.407 PMC5846057

[23] Sauerbrei W Taube SE McShane LM et al: Reporting recommendations for tumor marker prognostic studies (REMARK): An abridged explanation and elaboration. J Natl Cancer Inst 110:803–811 2018 29873743 10.1093/jnci/djy088 PMC6093349

[24] Caulley L Catalá-López F Whelan J et al: Reporting guidelines of health research studies are frequently used inappropriately. J Clin Epidemiol 122:87–94 2020 32184126 10.1016/j.jclinepi.2020.03.006

[25] da Costa BR Cevallos M Altman DG et al: Uses and misuses of the STROBE statement: Bibliographic study. BMJ Open 1:e000048 2011 10.1136/bmjopen-2010-000048 PMC3191404 22021739

[26] Sekula P Mallett S Altman DG et al: Did the reporting of prognostic studies of tumour markers improve since the introduction of REMARK guideline? A comparison of reporting in published articles. PLoS One 12:e0178531 2017 28614415 10.1371/journal.pone.0178531 PMC5470677

[27] ESMO: Young oncologist corner. https://www.esmo.org/career-development/young-oncologists-corner

[28] OncologyPro: ESMO handbook of interpreting oncological study publications. https://oncologypro.esmo.org/education-library/esmo-handbooks/interpreting-oncological-study-publications

[29] ASCO: https://www.asco.org/training-education/education-career-resources

[30] BMJ: Practical EBM tools. https://bestpractice.bmj.com/info/toolkit/ebm-toolbox

[31] BMJ: Critical appraisal checklists. https://bestpractice.bmj.com/info/toolkit/ebm-toolbox/critical-appraisal-checklists

[32] Lang TA Altman DG: Basic statistical reporting for articles published in biomedical journals: The “statistical analyses and methods in the published literature” or the SAMPL guidelines Smart P Maisonneuve H Polderman A Science Editors' Handbook. European Association of Science Editors 2013 10.1016/j.ijnurstu.2014.09.006 25441757

[33] Owens JK Nicoll LH: Plagiarism in predatory publications: A comparative study of three nursing journals. J Nurs Scholarsh 51:356–363 2019 30951246 10.1111/jnu.12475

[34] Ruiter-Lopez L Lopez-Leon S Forero DA: Predatory journals: Do not judge journals by their Editorial Board Members. Med Teach 41:691–696 2019 30794759 10.1080/0142159X.2018.1556390

[35] Singh Chawla D: Predatory-journal papers have little scientific impact. Nature 10.1038/d41586-020-00031-6 [epub ahead of print on January 13, 2020] 33432208

[36] Beall’s List: https://beallslist.net/

[37] National Institutes of Health: https://www.nihms.nih.gov/login/?next=/submission/

[38] Yamada Y: How to protect the credibility of articles published in predatory journals. Publications 9:4 2021

[39] Creative Commons: https://creativecommons.org/about/cclicenses

[40] Elsevier: https://scientific-publishing.webshop.elsevier.com/publication-process/difference-between-green-gold-open-access

[41] Whiffin CJ Smith BG Esene IN et al: Neurosurgeons' experiences of conducting and disseminating clinical research in low-income and middle-income countries: A reflexive thematic analysis. BMJ Open 11:e051806 2021 10.1136/bmjopen-2021-051806 PMC8461280 34551952

[42] Langer A Díaz-Olavarrieta C Berdichevsky K et al: Why is research from developing countries underrepresented in international health literature, and what can be done about it? Bull World Health Organ 82:802–803 2004 15643806 PMC2623037

[43] Doughty K Rothman L Johnston L et al: Low-income countries' orthopaedic information needs: Challenges and opportunities. Clin Orthop Relat Res 468:2598–2603 2010 20431972 10.1007/s11999-010-1365-x PMC3049618

[44] Nobes A Harris S: Open access in low- and middle-income countries: Attitudes and experiences of researchers [version 1; peer review: 2 approved with reservations]. Emerald Open Res 1:17 2019

[45] Till BM Rudolfson N Saluja S et al: Who is pirating medical literature? A bibliometric review of 28 million Sci-Hub downloads. Lancet Glob Health 7:e30–e31 2019 30554757 10.1016/S2214-109X(18)30388-7

[46] Bendezú-Quispe G Nieto-Gutiérrez W Pacheco-Mendoza J et al: Sci-Hub and medical practice: An ethical dilemma in Peru. Lancet Glob Health 4:e608 2016 27539805 10.1016/S2214-109X(16)30188-7

[47] Himmelstein DS Romero AR Levernier JG et al: Sci-Hub provides access to nearly all scholarly literature. Elife 7:e32822 2018 29424689 10.7554/eLife.32822 PMC5832410

[48] EndNote Click: https://kopernio.com/

[49] Hoy MB: Kopernio. J Med Libr Assoc 107:632–633 2019

[50] Hoy MB: New tools for finding full-text articles faster: Kopernio, nomad, unpaywall, and more. Med Ref Serv Q 38:287–292 2019 31379291 10.1080/02763869.2019.1629215

[51] Open Access Button: https://openaccessbutton.org/

[52] Unpaywall: https://unpaywall.org/

[53] Markham MJ Gentile D Graham DL: Social media for networking, professional development, and patient engagement. Am Soc Clin Oncol Ed Book 37:782–787 2017 10.1200/EDBK_180077 28561727

[54] Sedrak MS Attai DJ George K et al: Integrating social media in modern oncology practice and research. Am Soc Clin Oncol Ed Book 38:894–902 2018 10.1200/EDBK_204453 30231349

[55] ONCOLLEGE: https://oncollege.app/about-us

[56] Lengyel CG Altuna SC Habeeb BS et al: The potential of PI3K/AKT/mTOR signaling as a druggable target for endometrial and ovarian carcinomas. Curr Drug Targets 21:946–961 2020 31752654 10.2174/1389450120666191120123612

[57] Lengyel CG Habeeb B Khan SZ et al: Role of Her-2 in gastrointestinal tumours beyond gastric cancer: A tool for precision medicine. Gastrointest Disord 3:1–22 2021

[58] Lengyel CG Hussain S Trapani D et al: The emerging role of liquid biopsy in gastric cancer. J Clin Med 10:2108 2021 34068319 10.3390/jcm10102108 PMC8153353

[59] Trapani D Lengyel CG Habeeb BS et al: The global landscape of availability, accessibility and affordability of essential diagnostics and therapeutics for the management of HER2-positive breast cancer: The ONCOLLEGE-001 survey. J Cancer Policy 28:100285 2021 10.1016/j.jcpo.2021.100285 35559914

[60] El Bairi K Trapani D Petrillo A et al: Repurposing anticancer drugs for the management of COVID-19. Eur J Cancer 141:40–61 2020 33125946 10.1016/j.ejca.2020.09.014 PMC7508523

[61] TILS Breast Cancer: https://www.tilsinbreastcancer.org

[62] Dieci MV Radosevic-Robin N Fineberg S et al: Update on tumor-infiltrating lymphocytes (TILs) in breast cancer, including recommendations to assess TILs in residual disease after neoadjuvant therapy and in carcinoma in situ: A report of the International Immuno-Oncology Biomarker Working Group on Breast Cancer. Semin Cancer Biol 52:16–25 2018 29024776 10.1016/j.semcancer.2017.10.003

[63] Gonzalez-Ericsson PI Stovgaard ES Sua LF et al: The path to a better biomarker: Application of a risk management framework for the implementation of PD-L1 and TILs as immuno-oncology biomarkers in breast cancer clinical trials and daily practice. J Pathol 250:667–684 2020 32129476 10.1002/path.5406

[64] Hudeček J Voorwerk L van Seijen M et al: Application of a risk-management framework for integration of stromal tumor-infiltrating lymphocytes in clinical trials. NPJ Breast Cancer 6:15 2020 32436923 10.1038/s41523-020-0155-1 PMC7217941

[65] Kos Z Roblin E Kim RS et al: Pitfalls in assessing stromal tumor infiltrating lymphocytes (sTILs) in breast cancer. NPJ Breast Cancer 6:17 2020 32411819 10.1038/s41523-020-0156-0 PMC7217863

[66] Amgad M Stovgaard ES Balslev E et al: Report on computational assessment of tumor infiltrating lymphocytes from the International Immuno-Oncology Biomarker Working Group. NPJ Breast Cancer 6:16 2020 32411818 10.1038/s41523-020-0154-2 PMC7217824

[67] Long JC Hibbert P Braithwaite J: Structuring successful collaboration: A longitudinal social network analysis of a translational research network. Implement Sci 11:19 2016 26864452 10.1186/s13012-016-0381-y PMC4750242

[68] Steitz BD Levy MA: A social network analysis of cancer provider collaboration. AMIA Annu Symp Proc 2016:1987–1996 2017 28269958 PMC5333246

[69] Guyer RA Schwarze ML Gosain A et al: Top ten strategies to enhance grant-writing success. Surgery 10.1016/j.surg.2021.06.039 [epub ahead of print on July 19, 2021] PMC8642272 34294451

[70] Sudheesh K Duggappa DR Nethra SS: How to write a research proposal? Indian J Anaesth 60:631–634 2016 27729688 10.4103/0019-5049.190617 PMC5037942

[71] Sohn E: Secrets to writing a winning grant. Nature 577:133–135 2020 31863064 10.1038/d41586-019-03914-5

[72] Arthurs OJ: Think it through first: Questions to consider in writing a successful grant application. Pediatr Radiol 44:1507–1511 2014 25408132 10.1007/s00247-014-3053-6 PMC4236603

[73] Gemayel R Martin SJ: Writing a successful fellowship or grant application. FEBS J 284:3771–3777 2017 29154493 10.1111/febs.14318

[74] Wescott L Laskofski M: Grant writing tips for translational research. Methods Mol Biol 823:379–389 2012 22081359 10.1007/978-1-60327-216-2_25

[75] Brownson RC Colditz GA Dobbins M et al: Concocting that magic elixir: Successful grant application writing in dissemination and implementation research. Clin Transl Sci 8:710–716 2015 26577630 10.1111/cts.12356 PMC4739635

[76] Liu JC Pynnonen MA St John M et al: Grant-writing pearls and pitfalls: Maximizing funding opportunities. Otolaryngol Head Neck Surg 154:226–232 2016 26626133 10.1177/0194599815620174

[77] Inouye SK Fiellin DA: An evidence-based guide to writing grant proposals for clinical research. Ann Intern Med 142:274–282 2005 15710960 10.7326/0003-4819-142-4-200502150-00009

[78] Equator Network: https://www.equator-network.org/toolkits/writing-research

[79] Glonti K Cauchi D Cobo E et al: A scoping review on the roles and tasks of peer reviewers in the manuscript review process in biomedical journals. BMC Med 17:118 2019 31217033 10.1186/s12916-019-1347-0 PMC6585141

[80] Thistlethwaite J: Peer review: Purpose, process and publication. Clin Teach 9:201–204 2012 22783849 10.1111/j.1743-498X.2012.00612.x

[81] Clarivate: What happened to the Publons Academy? https://publons.com/academy/

[82] ecancer: https://ecancer.org

[83] ASCO Connection: COVID-19 disrupts critical career phase for early-career oncologists. https://connection.asco.org/blogs/covid-19-disrupts-critical-career-phase-early-career-oncologists

[84] Loriot Y Albiges-Sauvin L Dionysopoulos D et al: Why do residents choose the medical oncology specialty? Implications for future recruitment—Results of the 2007 French Association of Residents in Oncology (AERIO) Survey. Ann Oncol 21:161–165 2010 19628567 10.1093/annonc/mdp294

[85] UICC: Attracting medical students to the field of oncology. https://www.uicc.org/news/attracting-medical-students-field-oncology

